# Some of the Therapeutic Effects of Hydrochlorate of Ammonia

**Published:** 1880-12-20

**Authors:** Thomas S. Mitchell

**Affiliations:** Georgia


### SOME OF THE THERAPEUTIC EFFECTS OF HYDROCHLORATE OF AMMONIA.

BY THOMAS S. MITCHELL, M.D., OF GEORGIA.

*Case* 1. I was called, March 28th, 1877, to see Mrs. S.; found her suffering very much every catamenial period with severe "dragging down" pains in the back and hips; also from hemorrhoids. I have since operated for hemorrhoids, using the knife, with perfect success. The flow very small in quantity and in clots. She had borne two healthy children, the youngest eight years old. She has ever been a stout, healthy woman. I have known her from her early girlhood, attended her in the two confinements, which were natural and easy; was then suffering from indigestion, constipation, headache, etc.

I explained to her the trouble, and told her if she could bear more children she would get well again. She was willing to do anything to regain her lost health. I prescribed a digestive tonic with powder rhei for the constipation, and the muriate of ammonia in 10-grs. doses three times a day in water. Continued this line of treatment one month. She became pregnant; health restored almost entirely.

In April, 1878, she gave birth to a large healthy female child, and, strange to say, almost without a labor pain. She got up in the morning, got breakfast feeling unusually well. After attending to all her domestic duties, she sat down and said: "I think you had better send for Dr. M., I am in labor;" and in ten minutes more she gave birth to the child, sitting in the chair. I arrived in about one hour; found her quiet and without pain; delivered the placenta, and soon she was up at work without having a single pain or ache.

*Case.* 2. Was called to see Mrs. D.; found the symptoms and trouble nearly the same as Mrs. S.; her youngest child seven years old: had had two children.

I gave nearly the same treatment as I gave Mrs. S., and in a short time she became pregnant, and aborted at three months, with an alarming hemorrhage which was easy of control with fluid extract ergot, etc. Afterwards the ammonia treatment was continued. She became pregnant again, and with the constant use of the fluid ext. viburnum or cramp bark she went to full term and gave birth to a healthy living child.

